# A Natural Vibrio parahaemolyticus Δ*pirA*^*Vp*^ *pirB*^*Vp+*^ Mutant Kills Shrimp but Produces neither Pir^*Vp*^ Toxins nor Acute Hepatopancreatic Necrosis Disease Lesions

**DOI:** 10.1128/AEM.00680-17

**Published:** 2017-08-01

**Authors:** Kornsunee Phiwsaiya, Walaiporn Charoensapsri, Suwimon Taengphu, Ha T. Dong, Pakkakul Sangsuriya, Giang T. T. Nguyen, Hung Q. Pham, Piti Amparyup, Kallaya Sritunyalucksana, Suparat Taengchaiyaphum, Parin Chaivisuthangkura, Siwaporn Longyant, Paisarn Sithigorngul, Saengchan Senapin

**Affiliations:** a National Center for Genetic Engineering and Biotechnology, National Science and Technology Development Agency, Pathum Thani, Thailand; b Center of Excellence for Shrimp Molecular Biology and Biotechnology (Centex Shrimp), Faculty of Science, Mahidol University, Bangkok, Thailand; c Department Microbiology, Faculty of Science, King Mongkut's University of Technology Thonburi (KMUTT), Bangkok, Thailand; d Aquatic Molecular Genetics and Biotechnology Laboratory, National Center for Genetic Engineering and Biotechnology, National Science and Technology Development Agency, Pathum Thani, Thailand; e Institute of Aquaculture, Nha Trang University, Nha Trang, Vietnam; f Shrimp-Virus Interaction Laboratory, National Center for Genetic Engineering and Biotechnology, National Science and Technology Development Agency, Pathum Thani, Thailand; g Department of Biology, Faculty of Science, Srinakharinwirot University, Bangkok, Thailand; INRS—Institut Armand-Frappier

**Keywords:** Penaeus vannamei, shrimp, AHPND, EMS, Vibrio parahaemolyticus, Pir toxin

## Abstract

Acute hepatopancreatic necrosis disease (AHPND) of shrimp is caused by Vibrio parahaemolyticus isolates (VP_AHPND_ isolates) that harbor a pVA plasmid encoding toxins PirA^*Vp*^ and PirB^*Vp*^. These are released from VP_AHPND_ isolates that colonize the shrimp stomach and produce pathognomonic AHPND lesions (massive sloughing of hepatopancreatic tubule epithelial cells). PCR results indicated that V. parahaemolyticus isolate XN87 lacked *pirA*^*Vp*^ but carried *pirB*^*Vp*^. Unexpectedly, Western blot analysis of proteins from the culture broth of XN87 revealed the absence of both toxins, and the lack of PirB^*Vp*^ was further confirmed by enzyme-linked immunosorbent assay. However, shrimp immersion challenge with XN87 resulted in 47% mortality without AHPND lesions. Instead, lesions consisted of collapsed hepatopancreatic tubule epithelia. In contrast, control shrimp challenged with typical VP_AHPND_ isolate 5HP gave 90% mortality, accompanied by AHPND lesions. Sequence analysis revealed that the pVA plasmid of XN87 contained a mutated *pirA*^*Vp*^ gene interrupted by the out-of-frame insertion of a transposon gene fragment. The upstream region and the beginning of the original *pirA*^*Vp*^ gene remained intact, but the insertion caused a 2-base reading frameshift in the remainder of the *pirA*^*Vp*^ gene sequence and in the downstream *pirB*^*Vp*^ gene sequence. Reverse transcription-PCR and sequencing of 5HP revealed a bicistronic *pirAB*^*Vp*^ mRNA transcript that was not produced by XN87, explaining the absence of both toxins in its culture broth. However, the virulence of XN87 revealed that some V. parahaemolyticus isolates carrying mutant pVA plasmids that produce no Pir^*Vp*^ toxins can cause mortality in shrimp in ponds experiencing an outbreak of early mortality syndrome (EMS) but may not have been previously recognized to be AHPND related because they did not cause pathognomonic AHPND lesions.

**IMPORTANCE** Shrimp acute hepatopancreatic necrosis disease (AHPND) is caused by Vibrio parahaemolyticus isolates (VP_AHPND_ isolates) that harbor the pVA1 plasmid encoding toxins PirA^*Vp*^ and PirB^*Vp*^. The toxins are produced in the shrimp stomach but cause death by massive sloughing of hepatopancreatic tubule epithelial cells (pathognomonic AHPND lesions). V. parahaemolyticus isolate XN87 harbors a mutant pVA plasmid that produces no Pir toxins and does not cause AHPND lesions but still causes ∼50% shrimp mortality. Such isolates may cause a portion of the mortality in ponds experiencing an outbreak of EMS that is not ascribed to VP_AHPND_. Thus, they pose to shrimp farmers an additional threat that would be missed by current testing for VP_AHPND_. Moribund shrimp from ponds experiencing an outbreak of EMS that exhibit collapsed hepatopancreatic tubule epithelial cells can serve as indicators for the possible presence of such isolates, which can then be confirmed by additional PCR tests for the presence of a pVA plasmid.

## INTRODUCTION

A newly emerging shrimp disease called acute hepatopancreatic necrosis disease (AHPND) is the cause of a major proportion of shrimp disease outbreaks that occur within approximately 35 days after shrimp ponds are stocked. Currently, shrimp farmers collectively lump all such outbreaks under the term early mortality syndrome (EMS), mostly without verifying the cause. Thus, in technical reports, EMS should not be equated with AHPND (1). AHPND severely threatens the shrimp farming industry in Asia (including China, Vietnam, Malaysia, Thailand, and the Philippines) (1–5) but also that in Mexico (6, 7).

The pathognomonic lesions of AHPND are characterized by massive sloughing of tubule epithelial cells of the shrimp hepatopancreas (HP) in the absence of accompanying bacteria or other pathogens, suggesting the involvement of initially unidentified toxins (2). The causative agent of AHPND was eventually found to be unique isolates of Vibrio parahaemolyticus (VP_AHPND_ isolates) (3) that carry a plasmid (pVA1 or pVPA3-1) of approximately 69 kb carrying genes for toxins that resemble the binary Photorhabdus insect-related (Pir) toxins PirA and PirB (3, 8–11). These are released from VP_AHPND_ isolates that colonize the shrimp stomach (12) and enter the HP to produce pathognomonic AHPND lesions. Here we call them the PirA^*Vp*^ and PirB^*Vp*^ toxins to clearly indicate that they are Pir-like toxins in V. parahaemolyticus (Pir^*Vp*^) and not Pir toxins from Photorhabdus in V. parahaemolyticus. The results of bioassays using singly or combined heterologously expressed Pir^*Vp*^ toxins described in one publication indicated that both PirA^*Vp*^ and PirB^*Vp*^ are required to cause pathognomonic AHPND lesions in shrimp (13), while others have suggested that PirB^*Vp*^ alone (but not PirA^*Vp*^ alone) can do so (11).

Variations in virulence have also been reported among individual VP_AHPND_ isolates (4, 7, 14), and it has been suggested that the quantity of secreted toxins and not the plasmid copy number determines their virulence (14). A study of the sequence variation among the virulence plasmids of VP_AHPND_ isolates divided them into Southeast Asian types and Mexican types on the basis of the presence of a transposon (∼4 kb) and small repeat sequences of 9 bp (15). Additionally, the natural acquisition of pVA1 containing no *pir*^*Vp*^ toxin genes or their natural deletion from pVA1 has been suggested (11, 14). More recently, AHPND-causing species of Vibrio tentatively identified as V. harveyi (16) and identified as V. campbellii and V. owensii (17, 18) have also been found to harbor pVA virulence plasmids that are suspected to have been acquired by gene transfer from a VP_AHPND_ isolate. Here we describe the discovery and characterization of a virulent V. parahaemolyticus isolate, XN87, that was recovered from a shrimp pond in central Vietnam and that contained a mutant pVA plasmid with a mutant *pirA*^*Vp*^ gene but a normal *pirB*^*Vp*^ gene. However, we discovered that XN87 does not produce detectable levels of either of the Pir^*Vp*^ toxins, even though it still causes ∼50% shrimp mortality without pathognomonic AHPND lesions.

## RESULTS

### Unexpected PCR amplicons obtained using VP_AHPND_ detection methods.

**FIG 1:**
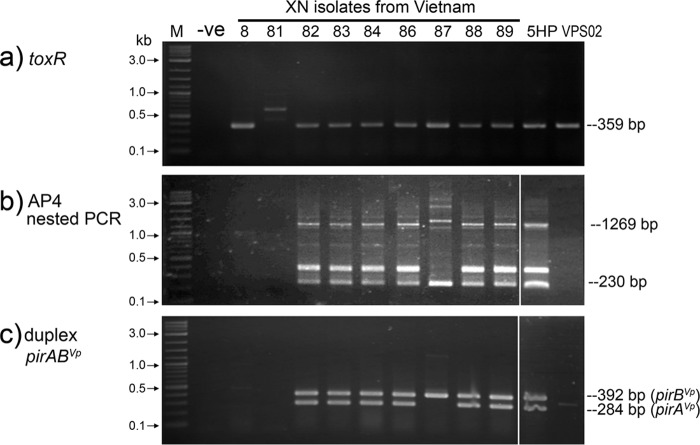
PCR detection assay results for Vibrio isolates from Vietnam and Thailand. Numbers represent individual bacterial isolates. V. parahaemolyticus 5HP (VP_AHPND_) and VPS02 (VP_non-AHPND_) from Thailand were included as positive and negative controls, respectively. (a) PCR amplicons for V. parahaemolyticus *toxR*; (b) AP4 nested PCR showing the expected AHPND results for all isolates except XN87; (c) duplex PCR for both the *pirA*^*Vp*^ and *pirB*^*Vp*^ genes showing the absence of *pirA*^*Vp*^ for XN87 only. Lanes M, DNA marker (2-log DNA ladder; New England BioLabs); lanes −ve, no-template control.

When tested by PCR for the presence of the V. parahaemolyticus species-specific *toxR* gene, isolates 5HP and VPS02 and all the Vibrio isolates from Vietnam except XN81 gave the expected 359-bp amplicon, indicating the presence of the *toxR* gene (Fig. 1a). Because XN81 gave a negative result by this test, it was excluded from further detailed study, although growth on thiosulfate-citrate-bile-sucrose (TCBS) agar suggested that it might be another Vibrio species.

Upon further testing using the AP4 PCR method to detect VP_AHPND_ isolates, isolate XN81 (already excluded from further detailed study) gave negative results, as expected, but so also did isolate XN8 (Fig. 1b), which was also excluded from further detailed study because it was a V. parahaemolyticus isolate that did not cause AHPND (VP_non-AHPND_ isolate). At the same time, the absence of both the *pirA*^*Vp*^ and *pirB*^*Vp*^ toxin genes from both of these isolates was confirmed by negative duplex PCR test results (Fig. 1c).

For the remaining 9 isolates, most gave the expected results using the AP4 nested PCR method. This consisted of no detectable bands from VP_non-AHPND_ isolate VPS02, while results for the remaining 8 V. parahaemolyticus isolates (Fig. 1b) revealed that only 7 (XN82, XN83, XN84, XN86, AN88, AN89, and 5HP) gave the expected amplicons for the AP4 primers (i.e., amplicons of 1,269 and 230 bp plus cross-primer products of 357 bp and 1,142 bp). The exception was isolate XN87, which gave unusual results, in that the first PCR amplicon was clearly larger than 1,269 bp and the additional amplicon of 357 bp was missing (Fig. 1b). Additionally, for XN87, the duplex PCR results in Fig. 1c revealed the presence of a *pirB*^*Vp*^ amplicon, while the expected amplicon for *pirA*^*Vp*^ was absent. To understand these results, some amplicons were subjected to sequence analysis.

### A transposase sequence was inserted into the *pirA*^*Vp*^ toxin gene of XN87.

**FIG 2:**
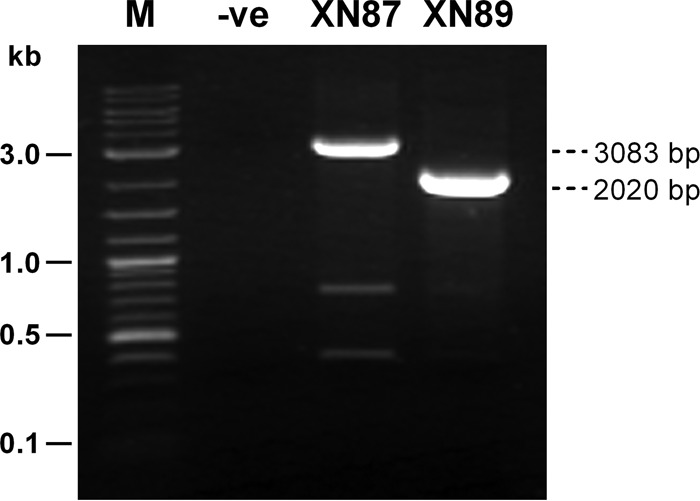
PCR amplicons obtained from XN87 and XN89 using primers PirAB-2020F/PirAB-2020R covering the whole region of the *pirA*^*Vp*^ and *pirB*^*Vp*^ genes. The amplicon from XN87 is approximately 1,000 bp larger than that from VP_AHPND_ isolate XN89. Lane M, DNA marker (2-log DNA ladder; New England BioLabs); lane −ve, no-template control.

**FIG 3:**
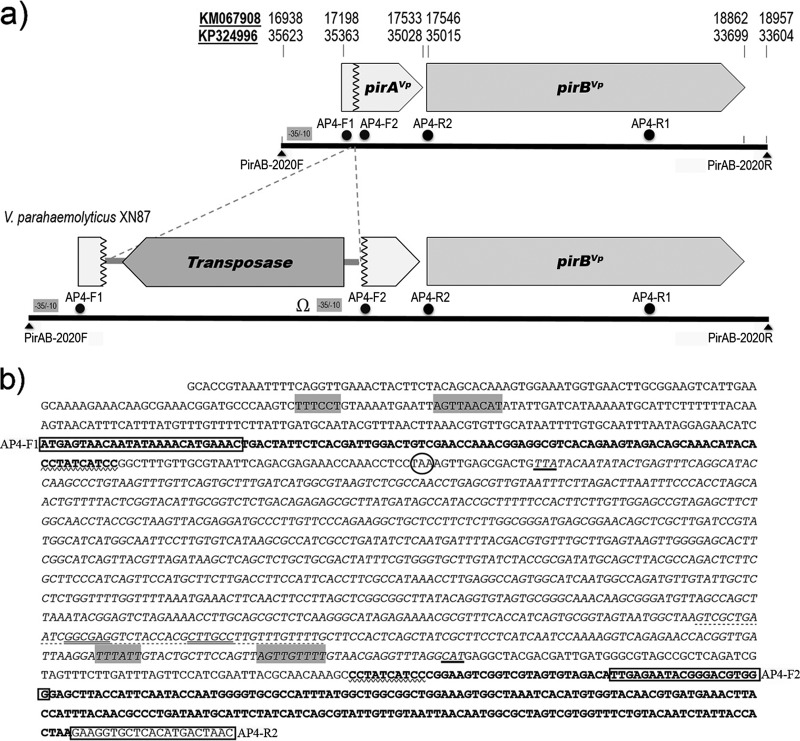
Comparison of the normal pVA1 plasmid and its mutated form in isolate XN87. (a) Scaled diagram of the *pirA*^*Vp*^ and *pirB*^*Vp*^ toxin gene region in the normal pVA1 plasmid and in the mutated plasmid of XN87 with a transposon gene fragment inserted into the *pirA*^*Vp*^ gene. Solid lines, PCR fragments of 2,020 bp and 3,083 bp amplified from the respective normal and mutant plasmids using primers PirAB-2020F and PirAB-2020R; dots, names and positions of the primers used for the AP4 nested PCR method for the detection of VP_AHPND_ isolates (see panel b for the primer sequences); gray boxes, potential −10 and −35 consensus sequences; Ω, a putative transcription termination sequence with a stem-loop structure. Numbers at the top of the diagram indicate nucleotide positions in the respective genome sequences of complete pVA1 or pVPA3-1 plasmids in the GenBank database (GenBank accession numbers KM067908 and KP324996). (b) Detailed sequence of a portion of the XN87 mutant plasmid derived from the PirAB-2020F to AP4-R2 primers showing where the *pirA*^*Vp*^ gene sequence (bold typeface) was interrupted by the transposon gene insertion (italic typeface) that also initiated a 2-base reading frameshift. Note that the last 10 nucleotides just before the start of the inserted transposable element (zigzag underlined bases CCTATCATCC) are repeated (also zigzag underlined) at the point of resumption of the *pirA*^*Vp*^ sequence (bold typeface). Circle, a potential stop codon shortly after the start of the insertion; bold underlines, the positions of the complementary start and stop codons for the inverted transposase gene sequence; gray-highlighted nucleotide sequences, potential −10 and −35 consensus sequences; dotted underline, a putative transcription termination sequence; double underline, base-pairing stem regions.

Sequence analysis to understand the reason for the unusual PCR amplicons from XN87 focused on comparison of its PCR amplicons with those from XN89 when using the AP4 primers (Fig. 1b) and the PirAB-2020F/PirAB-2020R primers (Fig. 2). Sequencing of the amplicons from XN89 (230, 1,269, and 2,020 bp) confirmed the presence of intact *pirA*^*Vp*^ and *pirB*^*Vp*^ toxin genes, with the sequences of all amplicons showing 100% identity to the sequences of the corresponding regions of the pVA1 or pVPA3-1 plasmids of VP_AHPND_ isolates recorded in the GenBank database (e.g., GenBank accession numbers KM067908 and KP324996) (Fig. 3a). The 230-bp amplicon generated using the AP4-F2 and AP4-R2 primers with isolate XN87 was also identical to that of XN89 and the GenBank records, indicating an intact junction between the *pirA*^*Vp*^ and *pirB*^*Vp*^ toxin genes. However, sequencing of the unusually large fragment (3,083 bp) amplified from isolate XN87 using the PirAB-2020F/PirAB-2020R primers (Fig. 2 and 3) revealed the presence of an out-of-frame insertion of 1,053 bp into the *pirA*^*Vp*^ gene adjacent to the decanucleotide CCTATCATCC in the *pirA*^*Vp*^ coding sequence (nucleotide positions corresponding to positions 17291 to 17300 of the sequence with GenBank accession number KM067908 or positions 35261 to 35270 of the sequence with GenBank accession number KP324996). This decanucleotide sequence was duplicated at the end of the insertion sequence (Fig. 3b). Thus, the downstream region of the original *pirA*^*Vp*^ gene beyond the inserted element, including the region covering the junction with the downstream *pirB*^*Vp*^ gene sequence, remained intact, except that these sequences were 2 bases out of frame due to the nature of the inserted sequence (Fig. 3b).

The inserted sequence resembled that of a transposable element reading in the direction opposite that of the *pirA*^*Vp*^ and *pirB*^*Vp*^ genes. It included 78 nucleotides of a 5′ untranslated region (UTR), a 921-nucleotide open reading frame (ORF), and a 54-nucleotide 3′ UTR (Fig. 3). The 921-bp ORF encoded a putative transposase protein of 306 deduced amino acids whose sequence was exactly identical to that of the translated transposase ORFs in the pVA1 or pVPA3-1 plasmids of previously reported VP_AHPND_ isolates (e.g., ORFs 18 and 26 of the sequence with GenBank accession number KM067908 and ORFs 15, 48, and 55 of the sequence with GenBank accession number KP324996).

A putative promoter region upstream of the *pirAB*^*Vp*^ binary toxin genes of XN89 and the mutated *pirAB*^*Vp*^ genes of XN87 containing a −10 box (AGTTAACAT) and a −35 box (TTTCCT) was predicted computationally (Fig. 3). Interestingly, the inverted transposase gene insertion created a putative transcription termination site potentially forming a stem-loop secondary structure (Fig. 3). It also included a potential promoter sequence upstream of the *pirB*^*Vp*^ gene of XN87 (Fig. 3).

### Reverse transcription (RT)-PCR revealed no bicistronic *pirAB*^*Vp*^ mRNA from XN87.

**FIG 4:**
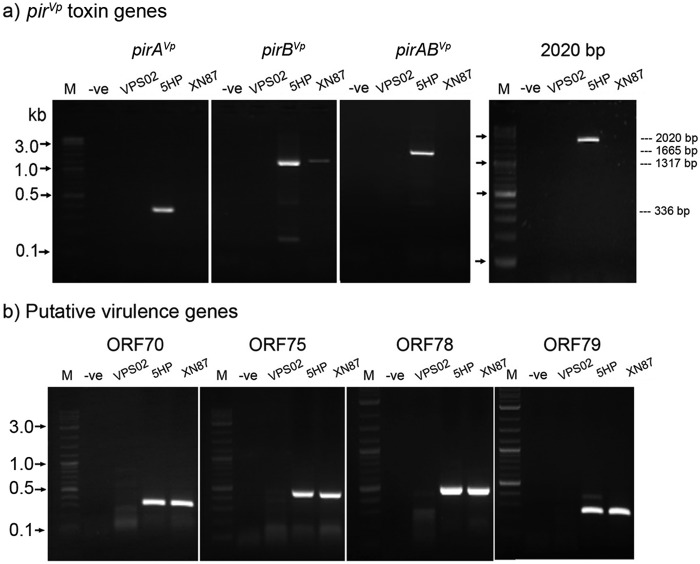
DNase-treated RNA extracts from the indicated V. parahaemolyticus isolates were assayed by RT-PCR using primers targeting Pir^*Vp*^ toxin genes (a) and putative virulence genes (b). Lanes M, DNA marker (2-log DNA ladder; New England BioLabs); lanes −ve, no-template control.

To test for the production of mRNA arising from the modified Pir^*Vp*^ binary toxin region of XN87, RT-PCR and sequencing of amplicons were carried out, and the results were compared to those obtained using the reference AHPND isolate 5HP. The results revealed that VP_AHPND_ 5HP produced a bicistronic mRNA, as evidenced by amplicons of 1,665 and 2,020 bp being obtained using the respective primers covering the region of the tandem *pirAB*^*Vp*^ ORFs, while no such detectable amplicons were obtained for XN87 and VPS02 (Fig. 4a). When RT-PCRs were carried out for ORFs *pirA*^*Vp*^ and *pirB*^*Vp*^ individually, very clear amplicons were obtained for both ORFs from control AHPND isolate 5HP, as expected (Fig. 4a). In contrast, the *pirA*^*Vp*^-interrupted XN87 isolate produced no amplicon for *pirA*^*Vp*^ but a low-intensity amplicon of 1,317 bp for *pirB*^*Vp*^ (Fig. 4a), possibly due to the incurred promoter sequence in the inserted transposon upstream of the *pirB*^*Vp*^ ORF (Fig. 3). Sequencing results for two individual recombinant clones revealed that the inserted sequence from one clone was identical to the sequence of *pirB*^*Vp*^ in the sequences with GenBank accession numbers KM067908 and KP324996, while the sequence of the other clone differed by 2 nucleotides. Sequences from both clones seemed to be translatable mRNAs since they did not contain any interrupted stop codons. The same batch of DNase-treated RNA was subjected to further transcriptional expression assays for selected putative virulence genes that were also located in the pVPA3-1 plasmid and the included ORFs 70, 75, 78, and 79 (encoding a trypsin family protein, a type II secretion protein, metalloprotease RseP, and type II and III secretion proteins, respectively; see Table 2 for the list of primers) (10). RT-PCR results (Fig. 4b) revealed the presence and similar expression levels of these target gene transcripts for 5HP and XN87, while they were absent from VP_non-AHPND_ isolate VPS02. The DNA sequences of the amplicons of XN87 were confirmed to be identical to the sequence with GenBank accession number KM067908. Additional PCR amplification tests using the DNase-treated RNA template material did not yield any products, confirming the absence of DNA contamination (see Fig. S1 in the supplemental material).

### XN87 produces no detectable Pir^*Vp*^ toxins in broth culture.

**FIG 5:**
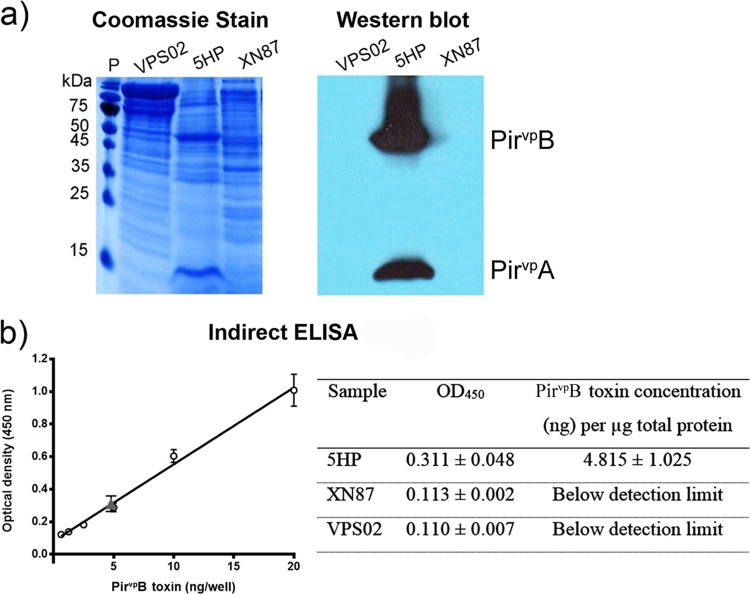
Expression and quantification of the Pir^*Vp*^ toxins. (a) Protein (20 μg) from the 80% ammonium sulfate precipitate fractions of the culture broths of the tested V. parahaemolyticus isolates was stained with Coomassie brilliant blue R-250 (left) and probed with monoclonal antibodies specific to the PirA^*Vp*^ and PirB^*Vp*^ proteins (right). Lane P, prestained protein marker (Thermo Scientific). (b) An indirect ELISA was performed to quantify the PirB^*Vp*^ protein from protein precipitates using serially diluted recombinant PirB^*Vp*^ toxin protein to make a standard curve (left). One microgram of the 80% protein fraction from each bacterial isolate was subjected to quantification, and the amounts of PirB^*Vp*^ are shown in the table (right). The gray triangle in the plot on the left represents the measurement for PirB^*Vp*^ in 5HP protein precipitates.

To test the ability of XN87 to produce Pir^*Vp*^ toxins, it was grown in broth culture and the broth was then subjected to protein fractionation by ammonium sulfate precipitation as previously described for isolation of the Pir^*Vp*^ toxins (13). The results showed the absence of protein bands and positive Western blotting results for both of the Pir^*Vp*^ toxins in the 80% ammonium sulfate precipitate from XN87 and VPS02 but the presence of both for positive-control isolate 5HP (Fig. 5a). Using serially diluted recombinant PirB^*Vp*^ (rPirB^*Vp*^) protein, the detection limit for an immunopositive dot blot assay result was found to be 0.004 to 0.008 ng (Fig. S2). By comparison to the Western blotting result for the detection of *pirB*^*Vp*^ in the 80% protein precipitate from 5HP, the lowest dilution that gave an immunopositive result was 0.002 to 0.004 μg total protein (Fig. S2). Thus, it could be concluded that the immunopositive signal from the crude protein preparation of 5HP indicated 0.004 to 0.008 ng PirB^*Vp*^ in 0.002 to 0.004 μg total protein, equal to 2 to 4 ng PirB^*Vp*^/μg total protein. In contrast, the Western blot for detection of the PirB^*Vp*^ protein in 20 μg the 80% protein precipitate from the broth of XN87 gave no detectable immunopositive signal, indicating that the quantity of PirB^*Vp*^, if any, was less than 2 to 4 ng/20 μg, which was equal to <0.1 to 0.2 ng/μg total protein. An indirect enzyme-linked immunosorbent assay (ELISA) was further employed to quantify the PirB^*Vp*^ toxin levels in the protein precipitates from 5HP and XN87. This revealed that 1 μg total protein from the 5HP precipitate contained 4.8 ± 1.0 ng of PirB^*Vp*^, while the amount of PirB^*Vp*^ in 1 μg the total protein precipitates from XN87 and VPS02 was below the ELISA detection limit (i.e., 0.3 ng of recombinant protein) (Fig. 5b). Taken together, the results confirmed that XN87 produced no detectable level of either Pir^*Vp*^ toxin in broth culture. Note that the immunopositive result for PirA^*Vp*^ at the lowest concentration of 0.078 μg total protein from 5HP indicated that the monoclonal antibody (MAb) specific for PirA^*Vp*^ was much less sensitive than the MAb specific for PirB^*Vp*^ (0.004 μg of total protein) (Fig. S2).

### Isolate XN87 kills shrimp but does not cause AHPND.

**FIG 6:**
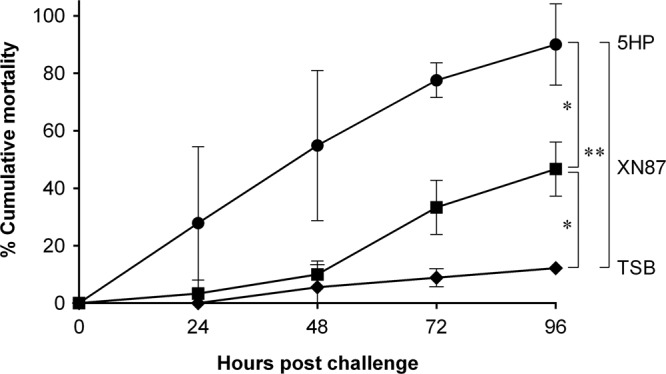
Cumulative percent mortality resulting from shrimp challenged with V. parahaemolyticus 5HP and XN87 isolates. The control group was mock challenged with TSB medium. Each group started with 9 to 15 P. vannamei shrimp, and experiments were conducted in duplicate. Statistical significance was calculated by one-way analysis of variance, and *P* values of <0.05 (*) and <0.01 (**) were considered significant.

The results from the two bioassays using isolates 5HP (VP_AHPND_) from Thailand (4) and XN87 from Vietnam revealed that 5HP gave a mean cumulative shrimp mortality at 96 h postchallenge of 90% (23/26 shrimp died) (Fig. 6), and this was significantly different (*P* < 0.05) from the mean cumulative mortality of 47% (14/30 shrimp died) over the same interval for XN87. At the same time, the mean mortalities for shrimp infected with both 5HP and XN87 were significantly different from the mean mortality for the control group (3/24 shrimp [13%] died) mock challenged with tryptic soy broth (TSB) medium (Fig. 6). Details of the mortality are given in Table S1.

**FIG 7:**
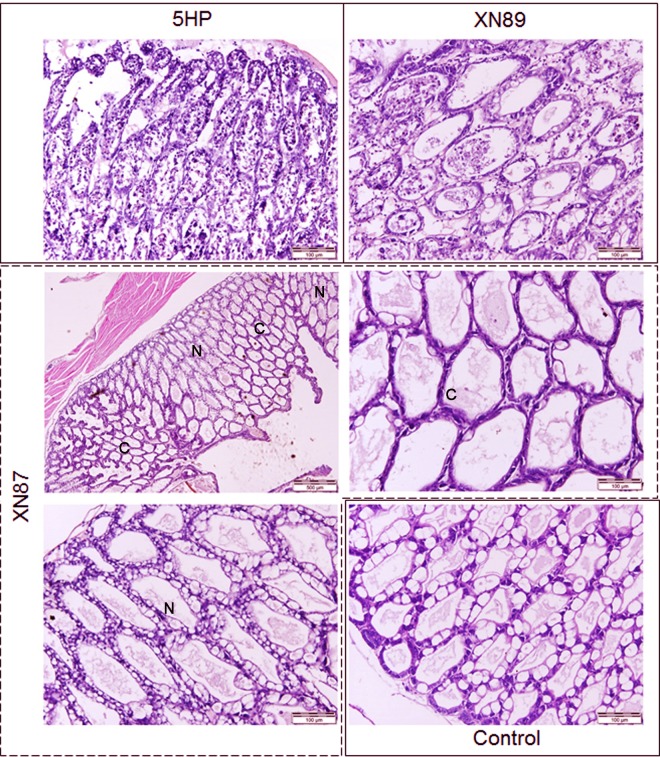
H&E-stained shrimp hepatopancreatic tissue from experimental challenge assays. Moribund shrimp from the groups immersed in 5HP and XN89 showed massive sloughing of hepatopancreatic tubule epithelial cells characteristic of AHPND. Samples from moribund shrimp exposed to XN87 showed a mixture of normal (N) and collapsed (C) hepatopancreatic tubule epithelia. The histology of the hepatopancreas was normal in survivors and moribund shrimp in the TSB and non-AHPND VPS02 immersion control groups.

Histological examination of moribund shrimp revealed that only those challenged with 5HP showed typical lesions pathognomonic for AHPND (i.e., massive hepatopancreatic epithelial cell sloughing into the lumen) (Fig. 7). In contrast, moribund shrimp from the immersion challenge with XN87 showed only collapsed hepatopancreatic tubule epithelial cells (approximately 50% of tubules in tissue sections) (Fig. 7). The morphology of hepatopancreatic tissue in the control groups treated with VPS02 or exposed to TSB medium only was normal (a representative normal HP is shown in Fig. 7).

## DISCUSSION

### Reasons for absence of detectable PirB^*Vp*^ toxin in XN87 culture broth.

Here we have characterized an isolate of V. parahaemolyticus (XN87) that harbors a mutant pVA plasmid with insertion of a transposon sequence into its *pirA*^*Vp*^ gene. By transcription and translation analysis, the mutant did not produce either bicistronic *pirAB*^*Vp*^ mRNA or the PirAB^*Vp*^ toxins, even though by RT-PCR it gave a faint amplicon for mRNA of the *pirB*^*Vp*^ gene. There are several possibilities as to why *pirB*^*Vp*^ mRNA might be present but not give rise to any detectable PirB^*Vp*^ toxin in the culture broth of XN87. One is the polar mutation effect, where mutations in polycistronic mRNA can result in a premature stop in translation (i.e., the stop codon described in Fig. 3) and lead to the reduction or prevention of expression of promoter-distal genes in the same operon (19, 20). Additionally, the occurrence of a putative transcriptional termination signal internally in the inverted transposon sequence might also cause interruption of the transcription of downstream sequences. It is also possible that the presence of a potential promoter sequence in the inverted transposon sequence might lead to a low level of expression of the PirB^*Vp*^ protein but at a level too low to be detectable by ELISA or Western blot assay. The effects of transposon insertion resulting in the inactivation of genes have been reported in several works (21–23).

Whatever the cause, the result from the Western blot assay using 20 μg of the protein precipitate from XN87 gave no detectable immunopositive signal, indicating that the quantity of PirB^*Vp*^, if any, was less than 2 to 4 ng/20 μg (<0.1 to 0.2 ng/μg) and was at least 20 times less than the 2 to 4 ng PirB^*Vp*^/μg total protein in the crude protein precipitate of 5HP. By reference to earlier work (13), this minimum detectable level is 25,000 to 50,000 times less than the 5 μg of heterologously expressed PirB^*Vp*^ (rPirB^*Vp*^) toxin that caused no mortality or abnormal histology in test shrimp by reverse gavage assay. Thus, we concluded that any possible level of PirB^*Vp*^ toxin that might have been produced by the faintly detectable RT-PCR amplicon for *pirB*^*Vp*^ mRNA in the broth precipitate of XN87 was physiologically negligible and could not in itself be responsible for shrimp mortality of approximately 50% accompanied by collapsed epithelia in the HP. This conclusion supported our proposal that the cause was other toxic factors produced by XN87. At the same time, the fact that protein expression by an intact *pirB*^*Vp*^ gene can be controlled to such a low level suggests that further investigation of the controls over Pir^*Vp*^ toxin expression might yield information that could be applied in controlling the pathogenicity of VP_AHPND_ isolates.

### Virulence of XN87.

In the introduction section, variation in the virulence of AHPND isolates was mentioned (4, 7, 14, 18), and even the most virulent VP_AHPND_ isolate used in this study (5HP) was apparently less virulent than several VP_AHPND_ isolates described from Mexico (7). XN87, which caused 47% mortality in 4 days, showed much less virulence than any of these AHPND isolates, but this level of mortality would still be a serious problem for a shrimp farmer. Thus, despite its inability to produce the PirA^*Vp*^ and PirB^*Vp*^ toxins or to cause AHPND lesions, it is important to understand the reason for its pathogenicity. The mortality caused by XN87 was accompanied by an abnormal HP histology in the form of collapsed tubule epithelial cells, and this was similar to the histopathology reported for moribund shrimp from immersion challenges with low concentrations of VP_AHPND_ isolates (4). In the same publication (4), a V. parahaemolyticus isolate (2HP) that did not cause AHPND did cause significant shrimp mortality (67%) similar to that caused by XN87, and the moribund shrimp also showed a histopathology characterized by collapsed HP tubule epithelia. They also reported that 2HP gave a positive test result with the AP2 detection method that we now know indicates the presence of a pVA plasmid, so it is possible that 2HP, too, may produce no PirA^*Vp*^ or PirB^*Vp*^ toxin. This isolate and XN87 differ from other more recently reported isolates of V. parahaemolyticus that carry genetically variable pVA plasmids, since they were apparently not virulent for shrimp and did not produce collapsed HP tubule epithelia (12, 18).

Genetic variation in the virulence plasmid of V. parahaemolyticus has so far been noted in five other studies (11, 14, 15, 17, 18). The natural V. parahaemolyticus strain M2-36, which lacks the entire *pirAB*^*Vp*^ operon, failed to cause AHPND (11). Similar results were reported by Han et al. (18) for Pir^*Vp*^ toxin-minus mutants. At the same time, Mexican V. parahaemolyticus isolate 13-306/D4, which contains a Tn*3*-like transposon insertion but also intact *pirAB*^*Vp*^ genes, caused 100% cumulative mortality, while strains with a transposon insertion but without the *pirAB*^*Vp*^ genes were nonpathogenic (15). V. parahaemolyticus strain E1M, which lacks a part of the *pirA*^*Vp*^ gene but which carries the entire *pirB*^*Vp*^ gene, did not kill shrimp (14). Two studies reported variable plasmid copy numbers from isolate to isolate (14, 15), and one of those studies indicated that virulence did not depend on plasmid copy number but depended on the quantity of secreted PirAB^*Vp*^ toxins (14).

The very recent publication by Han et al. (18) includes descriptions of six V. parahaemolyticus isolates carrying mutant pVA plasmids with deletions covering the region of the *pirA*^*Vp*^ and *pirB*^*Vp*^ genes that are part of a 5,535-bp composite transposon that they named Tn*6264*. It consists of 2 inverted repeats of the IS*Val1* mobile element plus the *pirAB*^*Vp*^ genes. Three of these isolates (called type I mutants) lacked both the *pirA*^*Vp*^ and *pirB*^*Vp*^ genes, while another three (called type II mutants) lacked only the *pirA*^*Vp*^ gene (i.e., they still carried an intact *pirB*^*Vp*^ gene). Another very recent publication (17) describes an isolate of V. owensii that causes AHPND and has a pVA plasmid that carries both the *pirA*^*Vp*^ and *pirB*^*Vp*^ genes. That article also presented the results of an extensive *in silico* analysis of pVA plasmids containing the composite transposon that the authors named *pirAB*-Tn*903* (which is identical to the Tn*6264* composite transposon described above) and of pVA-related plasmids in the V. harveyi clade (pVH). None of these mutants included one with a transposable element inserted within the *pirAB*^*Vp*^ gene region.

In addition to evidence regarding the genetic variation of VP_AHPND_ isolates and mutants, previously reported reverse gavage assays using purified, heterologously expressed PirA^*Vp*^ and PirB^*Vp*^ toxins at 5 μg each per gram of shrimp also caused collapsed HP tubule epithelia, while 10 μg each caused a typical AHPND histopathology (13). The results corresponded to those obtained using low concentrations of VP_AHPND_ isolates in immersion assays as described above and indicated that low toxin or pathogen levels can cause collapsed HP tubule epithelia. Thus, it appears that the case definition for AHPND may need to be expanded to include the possibility of collapsed HP tubule epithelia as the indicator of the presence of VP_AHPND_ isolates, perhaps including V. parahaemolyticus isolates with mutated pVA plasmids. The problem is that collapsed HP tubule epithelia may result from many causes (even starvation) and are thus of little diagnostic value.

Another interesting phenomenon is that only 1 μg total protein per g shrimp (1 μg/g) from the 60% ammonium sulfate protein precipitate fraction of the culture broth from typical VP_AHPND_ isolate 5HP was able to cause a full-blown AHPND pathology, while 20 μg/g of the mixed, heterologously expressed toxins was needed to obtain the same effect. This suggested, in turn, that other proteins in that crude precipitate potentiated the effect of the PirA^*Vp*^ and PirB^*Vp*^ toxins (13). It also suggested that mortality in the bioassay with XN87, which carries a pVA plasmid but produces no Pir^*Vp*^ toxins, arose from a substance or substances that can cause shrimp mortality in its or their own right. Possibilities include other putative virulence genes that are located in the pVA plasmid and that were shown by our expression analysis to be present in XN87 but whose expression was unaffected by the loss of the Pir^*Vp*^ toxins.

In summary, mortality accompanied by collapsed HP tubule epithelia is common for (i) shrimp infected with XN87, which does not produce PirAB^*Vp*^ toxins but does produce other potential toxins; (ii) shrimp infected with low concentrations of VP_AHPND_ isolates in immersion bioassays; and (iii) shrimp exposed to low levels of mixed PirA^*Vp*^ and PirB^*Vp*^ toxins. These facts suggest that other toxins may potentiate the effects of the PirAB^*Vp*^ toxins and even raise the question as to whether or not they might be able, at a sufficient concentration alone, to cause the typical AHPND pathology. Whether an AHPND-potentiating toxin(s) originates from the pVA plasmid or from one or both of the host chromosomes is an open question. So is the matter of control over their expression. Isolate XN87 and perhaps also isolate 2HP provide an opportunity to answer these questions by focusing on the nature and function of other proteins that are present in their culture broth precipitates and how they compare to those present in the precipitates of VP_AHPND_ isolates, such as 5HP.

One prominent difference between our work and recently published work describing other bacterial isolates containing mutated pVA-related plasmids (18) is that isolates XN87 (described herein) and 2HP (described by Joshi et al. [4]) caused significant shrimp mortality, while mutants that contained pVA plasmids but that lacked the *pirAB*^*Vp*^ toxin genes described in the other reports caused no mortality. We currently have no firm explanation for this difference, but the bioassays by Han et al. (18) were carried out using bacteria added to feed pellets, while our tests were carried out by immersion. Differences in the results may also be related to individual characteristics of the isolates used. For example, two recent studies from Thailand revealed that isolates of V. parahaemolyticus positive for the pVA plasmid comprised a variety of serotypes (24, 25). In another study on the pathogenesis of VP_AHPND_ isolates (12), immersion challenges with isolate M1-1, which carried the pVA plasmid containing the *pirAB*^*Vp*^ toxin genes, gave negligible mortality at 96 h. Differences in virulence might be clarified upon sequencing and comparison of whole genomes for the presence and location of other factors that may control virulence.

In conclusion, the discovery of mutant isolate XN87 adds to the growing list of bacterial isolates that carry variants of the pVA plasmid and that have been described in previous publications. However, it also reveals the possibility that isolates of V. parahaemolyticus and perhaps other Vibrio species carrying mutated versions of pVA may still be virulent for shrimp, despite their inability to produce the PirAB^*Vp*^ toxins. Our work suggests that such isolates cause a portion of the mortality in ponds where shrimp experience EMS that cannot be ascribed to VP_AHPND_ isolates on the basis of histological analysis (see the introduction for the distinction between EMS and AHPND). Moribund shrimp exhibiting HP tissue with collapsed tubule epithelial cells in the absence of bacteria can be used to serve as a preliminary indicator for the possible presence of such isolates. A suspected presence could then be confirmed by additional PCR tests for the presence of a pVA plasmid, despite negative PCR test results for the presence of the *pirAB*^*Vp*^ toxin genes.

## MATERIALS AND METHODS

### Vibrio parahaemolyticus isolates and DNA extraction.

A total of 11 Vibrio isolates were used in the present study. Of these, 9 (XN8, XN81, XN82, XN83, XN84, XN86, XN87, XN88, and XN89) were isolated from shrimp at 50 to 60 days after stocking of Penaeus vannamei in different ponds in Ninh Thuan Province, Vietnam, in 2014. Some ponds had previously experienced EMS outbreaks in 2012 and 2013. The shrimp exhibited a shrunken hepatopancreas and slow growth. These isolates were selected because all gave green colonies on TCBS agar plates and were thus suitable for screening as potential VP_AHPND_ isolates. VP_AHPND_ isolate 5HP and non-AHPND isolate VPS02 served as positive and negative AHPND controls, respectively, and were available at the Center of Excellence for Shrimp Molecular Biology and Biotechnology (Centex Shrimp) (4). Details of the bacterial isolates used in this study are given in Table 1.

**TABLE 1:** Bacterial isolates used in this study

Isolate(s)	Description	Source	Province, country, yr of isolation	Reference or source
XN8	Green colony on TCBS, *toxR* positive, AP4 negative	P. vannamei	Ninh Thuan, Vietnam, 2014	This study
XN81	Green colony on TCBS, *toxR* negative, AP4 negative	P. vannamei	Ninh Thuan, Vietnam, 2014	This study
XN82, XN83, XN84, XN86, XN87, XN88, XN89	Green colony on TCBS, *toxR* positive, AP4 positive	P. vannamei	Ninh Thuan, Vietnam, 2014	This study
5HP	Green colony on TCBS, *toxR* positive, AP4 positive	P. vannamei from pond with EMS	Prachuap Khiri Khan, Thailand, 2012	4
VPS02	Green colony on TCBS, *toxR* positive, AP4 negative	Shrimp pond sediment	Phang Nga, Thailand, 2008	4

Bacterial DNA extraction was carried out using a heat extraction method. Briefly, bacterial cell pellets obtained by centrifugation of 3-ml overnight cultures were washed once with sterile distilled water, centrifuged, and resuspended in 200 μl of fresh sterile distilled water. The suspension was heated at 95°C for 10 min and quickly transferred to ice for 10 min. After centrifugation, the supernatant containing bacterial DNA was collected and the DNA concentration was measured by spectrophotometric analysis.

### PCR amplification methods.

The 9 isolates from Vietnam were first screened for the presence of the *toxR* gene (considered to be a species-specific marker for V. parahaemolyticus) using a previously published method (26), except that the forward primer was changed to toxR-F (Table 2). DNA extracts from 5HP and VPS02 were used as positive controls. Isolates that failed this test were excluded from further detailed study. The isolates and controls were also tested for AHPND toxin genes according to previously published methods (10, 27, 28). In addition, primers PirAB-2020F/PirAB-2020FR (Table 2) were used for amplification of a target region that included the tandem AHPND toxin genes (i.e., *pirA*^*Vp*^ and *pirB*^*Vp*^) and a portion of their upstream and downstream regions. PCR mixtures of 25 μl contained 200 ng of bacterial DNA, 0.2 μM each primer, 0.1 mM deoxynucleoside triphosphates, 1 mM MgCl_2_, 1 × reaction buffer, and 2.5 U of Platinum *Taq* DNA polymerase (Invitrogen). Thermal cycling condition consisted of 1 cycle of 94°C for 5 min, 25 cycles (*toxR*) or 35 cycles (*pirAB*^*Vp*^) of 94°C for 40 s, 50°C for 40 s, and 72°C for 30 s (*toxR*) or 2 min (*pirAB*^*Vp*^), and a final cycle of 72°C for 5 min. PCR products were analyzed by 1.5% agarose gel electrophoresis.

**TABLE 2:** Primers used in this study

Primer name	Primer sequence (5′-3′)	Target	Product size (bp)	Reference or source
toxR-F	ACGCAATCGTTGAACCAGAA	V. parahaemolyticus toxR	359	This study
toxR-R	ATACGAGTGGTTGCTGTCATG			26
PirAB-2020F	GCACCGTAAATTTTCAGGTT	Tandem *pirAB^Vp^* genes and a portion of their upstream and downstream regions	2,020	This study
PirAB-2020R	CGTTGCAATCTAAGACATAG			
VpPirA-F	ATGAGTAACAATATAAAAC	*pirA^Vp^* ORF	336	This study
VpPirA-R	TTAGTGGTAATAGATTG			
VpPirB-F	ATGACTAACGAATACGT	*pirB^Vp^* ORF	1,317	This study
VpPirB-R	CTACTTTTCTGTACCAA			
VpPirA-F and VpPirB-R		Tandem *pirAB^Vp^* ORFs	1,665	This study
VpORF70-F	CGTTGGCATCGTTCGTGTTG	ORF70 trypsin family protein	326	This study
VpORF70-R	TACCGTAAGTAGAGCGTTCA			
VpORF75-F	CCGATACAAACTCACCACCA	ORF75 type II secretion protein	429	This study
VpORF75-R	AGGAGAAGTAGGGCAAAGAC			
VpORF78-F	GATTGGCGTCAGTCAGTTCA	ORF78 metalloprotease RseP	452	This study
VpORF78-R	GCATCGCTTTGCTTTCTTTG			
VpORF79-F	CGCCGCCTGATTGACACCTA	ORF79 type II and III secretion protein	232	This study
VpORF79-R	TGCCATCCCATTTCCCCGTA			

### RT-PCR amplification methods.

For RT-PCR assay, 3 ml of an overnight broth culture of VPS02, 5HP, and XN87 was subjected to total RNA extraction using the TRIzol reagent (Invitrogen), and the RNA obtained was then treated with DNase I (Fermentas) to eliminate contaminating DNA. DNase-treated RNA (400 to 800 ng) was used as the template in RT-PCR mixtures containing 0.2 μM each primer targeting *pirAB*^*Vp*^ or other putative virulence genes (the primers are listed in Table 2), 1 μl of SuperScript one-step reverse transcriptase-Platinum *Taq* mix (Invitrogen), and 1× reaction buffer in a 25-μl volume. RT reactions were carried out at 50°C for 30 min. After the subsequent denaturation step of 94°C for 2 min, the reactions proceeded into PCR thermocycling, as described above under “PCR amplification methods,” except that the extension time at 72°C was adjusted to 1 min/kb. Additionally, the template RNA was subjected to PCR analysis using an established *pirAB*^*Vp*^ duplex PCR protocol (10) to ensure that the RNA used did not contain any contaminating DNA for the targeted toxins.

### DNA sequence analysis.

Selected PCR and RT-PCR amplicon bands were gel purified using a Favorgen gel/PCR purification kit and sent for direct sequencing with their respective primers. Some amplified products were cloned into the pGEM-T Easy cloning vector (Promega) prior to sequencing. Amplified fragments and recombinant plasmids were sequenced by 1st BASE Pte Ltd. (Malaysia). Sequence homology searches, comparisons, and translations were carried out using the BLAST, ClustalW, and ExPASy web tools, respectively. Putative promoter regions and transcription termination sites were predicted by the BPROM (29) and ARNold (30) web tools, respectively.

### Detection and analysis of PirAB^*Vp*^ in culture broth of bacterial isolates. (i) Western blot analysis.

Isolates 5HP, XN87, and VPS02 were grown in broth culture for preparation and analysis of dialyzed 80% ammonium sulfate precipitates by 12.5% SDS-PAGE by methods similar to those described in a previously published report (13). SDS-polyacrylamide gels were also subjected to Western blot analysis using mouse monoclonal antibodies specific for detection of the PirA^*Vp*^ and PirB^*Vp*^ toxins (31). Briefly, monoclonal antibodies against PirA^*Vp*^ or PirB^*Vp*^ were produced from mice immunized with recombinant proteins prepared as described previously (13). Spleen cell fusion with P3X myeloma cells was conducted using the method developed by Köhler and Milstein (32) with the modifications described by Mosmann et al. (33). Positive hybridoma clones were selected by dot blotting and Western blotting with recombinant PirA^*Vp*^ or PirB^*Vp*^ toxin proteins and subsequently cloned by limiting dilution. Hybridoma supernatants containing monoclonal antibodies were harvested and purified using a protein G affinity column (Roche). Purified antibodies were dialyzed, quantified, and stored at −20°C. The blots were incubated with a 1:3,000 dilution of each PirA^*Vp*^- or PirB^*Vp*^-specific monoclonal antibody. Goat anti-mouse immunoglobulin with a horseradish peroxidase (HRP)-conjugated secondary antibody was used, and detection was performed using a chemiluminescent substrate (Advansta), followed by X-ray film exposure. The detection sensitivity of the Western blotting assay was tested using serially diluted recombinant PirB^*Vp*^ (rPirB^*Vp*^) protein, which was obtained from previous work (13) and probed with anti-PirB^*Vp*^ antibody. Both monoclonal antibodies (MAbs) were used to analyze dialyzed 80% ammonium sulfate precipitates from bacterial culture broths for the presence of PirA^*Vp*^ and PirB^*Vp*^.

### (ii) Indirect ELISA detection of PirB^*Vp*^ toxin.

The dialyzed 80% ammonium sulfate precipitates from broth cultures of isolates 5HP, XN87, and VPS02 mentioned above were also subjected to ELISA for quantification of the PirB^*Vp*^ toxin. The purified rPirB^*Vp*^ protein was used to construct a standard curve. Each well of a 96-well plate was coated with 200 μl of coating buffer (0.05 M carbonate buffer, pH 9.6) containing serially diluted standard protein and exposed to the antibody overnight at 4°C. The plates were washed three times with washing buffer (1× phosphate-buffered saline [PBS] containing 0.05% [vol/vol] Tween 20, pH 7.4) and then blocked for 15 min at room temperature with 300 μl of blocking buffer (3% [wt/vol] bovine serum albumin in 1× PBS, pH 7.4). After the washing steps, the plates were incubated with the anti-PirB^*Vp*^ MAb followed by goat anti-mouse immunoglobulin with an HRP-conjugated secondary. Bound antibodies were detected by adding 3,3′,5,5′-tetramethylbenzidine (TMB) substrate (Sigma), and the reactions were stopped by adding 2 N H_2_SO_4_. The optical density at 450 nm (OD_450_) was measured using a microplate reader (SpectraMax).

### Shrimp bioassays to test bacteria for ability to cause AHPND.

Specific-pathogen-free (SPF) P. vannamei shrimp (body weight, 1.87 ± 0.2 g), kindly provided by the Charoen Pokphand Company, were used in experimental challenge tests. When this work was done, the Ethical Principles and Guidelines for the Use of Animals of the National Research Council of Thailand (1999) applied to vertebrates only and there was no official standard for invertebrates. However, its principles were adapted to shrimp. The guidelines of the New South Wales (Australia) state government for the humane harvesting of fish and crustaceans were also followed (http://www.dpi.nsw.gov.au/agriculture/livestock/animal-welfare/general/fish/shellfish) with respect to details regarding the transport of the shrimp and their laboratory maintenance. With respect to processing of the shrimp for histological analysis or for killing at the end of an experiment, the saltwater/ice slurry method was used as recommended in the Australian guidelines. The shrimp were acclimatized in the laboratory for 7 days before the experiments began. The bioassay was carried out twice. In the first bioassay, the shrimp were divided into 3 groups of 15 shrimp each in 20 liters continuously aerated artificial seawater at 20 ppt and ∼27°C. Isolates 5HP (positive-control isolate) and XN87 (test isolate) were first grown in tryptic soy broth (TSB) containing 1.5% (wt/vol) added NaCl at 30°C for 1 h with shaking. After dilution to 1:100 with fresh TSB medium, the bacterial cells were further cultured until the OD_600_ reached 0.6 (i.e., approximately 1 × 10^8^ CFU/ml, as verified by the plate count method). This inoculum was diluted appropriately in artificial seawater to obtain a final concentration of 10^6^ CFU/ml before the shrimp were added for the immersion challenges and maintained for the entire experiment of 96 h (4 days). For the negative-control shrimp group, TSB medium was added instead of the bacterial suspension. In the second bioassay, shrimp of the same size as the shrimp in the first assay were used and were tested according to the same protocol, except that the negative-control group contained only 9 shrimp, the positive-control group contained 11 shrimp, and the test group contained 15 shrimp, due to limitations in shrimp supply. Shrimp mortality was recorded daily, and moribund shrimp were fixed with Davidson's fixative solution for histopathological examination. Percent cumulative mortality was calculated for each bioassay, followed by preparation of a graph showing the mean percent mortality for the two bioassays with standard deviation bars. Statistical calculations were carried out using SPSS software, and differences were considered significant at a *P* value of ≤0.05. Dead and moribund shrimp were removed from the bioassay tanks as soon as they were noticed. The dead shrimp were discarded, while moribund shrimp and healthy control shrimp at 4 days were fixed and processed for histological analysis as described below.

### Histological examination.

Shrimp specimens were preserved with Davidson's fixative and processed for standard paraffin sectioning and hematoxylin and eosin (H&E) staining as described by Bell and Lightner (34). The slides were examined by light microscopy for abnormalities, including the presence of pathognomonic AHPND lesions, characterized by massive sloughing of hepatopancreatic tubule epithelial cells in the absence of bacteria (3).

### Accession number(s).

The GenBank accession numbers for the sequences reported in this paper are KU145395 to KU145400.

## Supplementary Material

Supplemental material
