# Supplementary material for: A Natural Vibrio parahaemolyticus ΔpirAVp pirBVp+ Mutant Kills Shrimp but Produces neither PirVp Toxins nor Acute Hepatopancreatic Necrosis Disease Lesions
Source: Appl Environ Microbiol. 2017 Aug 1;83(16):e00680-17. doi: 10.1128/AEM.00680-17 (PMC5541212; doi:10.1128/AEM.00680-17)
Supplement: Supplemental material [file supp_83_16_e00680-17__index.html]

A Natural Vibrio parahaemolyticus ΔpirAVp pirBVp+ Mutant Kills Shrimp but Produces neither PirVp Toxins nor Acute Hepatopancreatic Necrosis Disease Lesions — Supplemental material 

# A Natural Vibrio parahaemolyticus Δ*pirA**Vp* *pirB**Vp+* Mutant Kills Shrimp but Produces neither Pir*Vp* Toxins nor Acute Hepatopancreatic Necrosis Disease Lesions

## Supplemental material

- Supplemental file 1 -

  RT-PCR and PCR assays of DNase-treated RNAs from indicated *V. parahaemolyticus* isolates, using duplex primers targeting *pirAVp* and *pirBVp* genes (Fig. S1); sensitivity of the Western blot assay using serially diluted total protein from the 80% ammonium sulfate precipitate fraction from 5HP and recombinant PirB*Vp* (rPirB*Vp*); details of the cumulative mortality of *P. vannamei* challenged with *V. parahaemolyticus* 5HP and XN87 isolates (Table S1).

  PDF, 554K
