# Supplementary material for: A Natural Vibrio parahaemolyticus ΔpirAVp pirBVp+ Mutant Kills Shrimp but Produces neither PirVp Toxins nor Acute Hepatopancreatic Necrosis Disease Lesions
Source: Appl Environ Microbiol. 2017 Aug 1;83(16):e00680-17. doi: 10.1128/AEM.00680-17 (PMC5541212; doi:10.1128/AEM.00680-17)
Supplement: Supplemental material [file AEM.00680-17_zam999117977s1.pdf]

Supplemental Figure

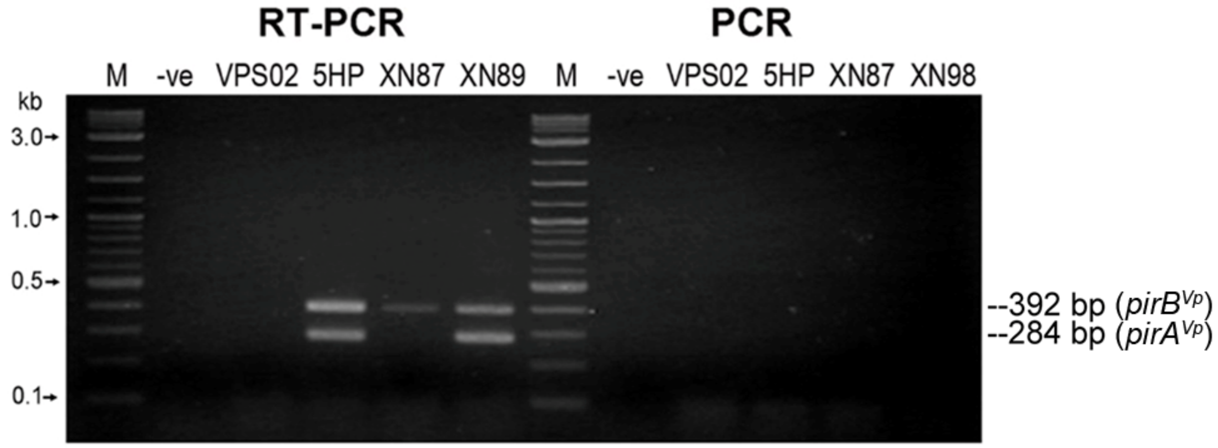

Figure S1. DNase-treated RNAs from indicated *V. parahaemolyticus* isolates were assayed by RT-PCR and PCR using duplex primers targeting *pirA*^{Vp} and *pirB*^{Vp} genes. No amplicons were obtained from PCR reactions confirming that there was no DNA contamination in the RT-PCR reactions. M, DNA marker, –ve, no template control.

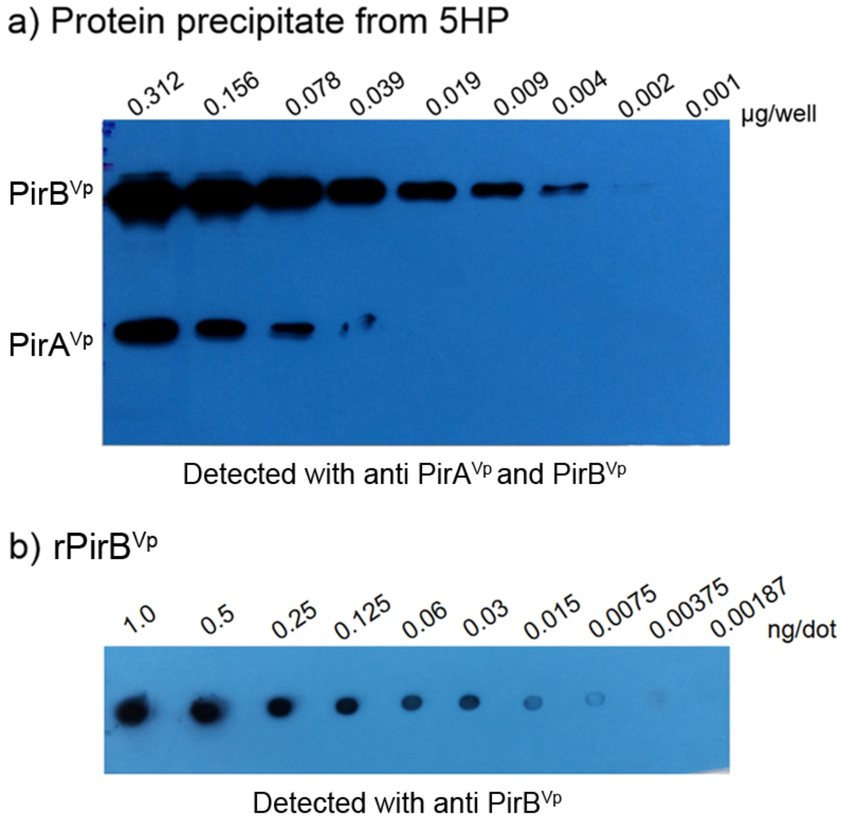

Figure S2. Sensitivity of the western blot assay using serially diluted total protein from the 80% ammonium precipitate fraction from 5HP and recombinant PirB^{Vp} (rPirB^{Vp}). (a) Western blot of total protein from 5HP probed with MAb against PirA^{Vp} and PirB^{Vp} and showing immunopositive results for PirA^{Vp} at 0.078 µg total protein and for PirB^{Vp} at 0.002-0.004 µg total protein. (b) Immunodot blot of serially diluted rPirB^{Vp} probed with MAb against PirB^{Vp} and showing immunopositive results for PirB^{Vp} at 0.004-0.008 µg. Thus, it can be concluded that the immunopositive signals for the lowest detectable concentration of PirB^{Vp} represent in the crude protein preparation of 5HP in (a) is 0.004-0.008 ng/0.002-0.004 µg total protein = 0.002-0.004 ng/µg total protein.

Supplemental Table

Table S1 Details of the cumulative mortality of *P. vannamei* challenged with *V. parahaemolyticus* 5HP and XN87 isolates.

**Hours post challenge**	**Cumulative mortality of *P. vannamei***					
	**TSB**		**5HP**		**XN87**	
	***n* = 15**	***n* = 9**	***n* = 15**	***n* = 11**	***n* = 15**	***n* = 15**
0	0	0	0	0	0	0
24	0	0	7	1	0	1
48	0	1	11	4	1	2
72	1	1	11	9	4	6
96	2	1	12	11	8	6
